# Supplementary material for: Model Re-Estimation: An Alternative for Poor Predictive Performance during External Evaluations? Example of Gentamicin in Critically Ill Patients
Source: Pharmaceutics. 2022 Jul 7;14(7):1426. doi: 10.3390/pharmaceutics14071426 (PMC9315759; doi:10.3390/pharmaceutics14071426)
Supplement: Supplementary file 1 [file pharmaceutics-14-01426-s001.zip › pharmaceutics-1717133-supplementary.pdf]

Supplementary Information

Table S1. Summary of the characteristics of the evaluated PopPK models.

Model	Cmpt	Cov	Error	θCL	IIV_{CL} (%)	θV or V_1	$IIV_{V\ or\ V1}$ (%)	θQ	IIV_Q (%)	θV_2	IIV_{V2} (%)	Residual variability		Model equations
												Proportional (%)	Additive (mg/L)	
Rea RS et al. [13]	1	GFR, BW	Mixed	3.14	83.7	53	64.4	-	-	-	-	24.3	0.381	$CL = (3.14 \times GFR^{1.2}) / (54.8^{1.2} + GFR^{1.2})$<br>$V_1 = 53 \times (BW/70)$
Bos JC et al.[14]	1	CrCl	Mixed	5.7	74	19	49	-	-	-	-	32	0.056	$CL = 5.7 \times (1 + 0.0091 \times (CrCl - 74))$
Hodiamont et al.[15]	2	IBW, ALBM	Proportional	1.15	42.5	21.2	17.2	1.96	-	18.4	-			$CL = 1.15 \times (IBW/70)^{0.75}$<br>$V_1 = 21.2 \times (IBW/70) \times (ALBM/22)^{-0.833}$
Hodiamont et al. [16]	2	-	Mixed	2.3	75.0	21.6	27.0	1.3	-	10.2	-	19.4	0.13	$CL = 2.3 \times (BW/70)^{0.75}$<br>$Q = 1.3 \times (BW/70)^{0.75}$<br>$V_1 = 21.6 \times (BW/70)$

Cmpt, compartment; *Cov*, covariates, *CL*, clearance; *V*, central volume of distribution; V_1, volume of distribution of the first compartment; *Q*, inter-compartmental clearance; V_2, volume of distribution of the second compartment; *IIV*, interindividual variability, *ALBM*, Albumin, *CrCl*, creatinine clearance; *GFR*, glomerular filtration rate, *BW*, bodyweight, *IBW*, ideal bodyweight.

Table S2. Summary of the characteristics of the re-estimated PopPK models.

Model	Cmpt	Cov	Error	θCL	IIV_{CL} (%)	θV or V_1	$IIV_{V\ or\ V1}$ (%)	θQ	IIV_Q (%)	θV_2	IIV_{V2} (%)	Model equations
Rea RS et al. [13]	1	GFR, BW	Mixed	9.31[a] 129[b]	36.9[a] 18.3[b]	21.7	28.8	-	-	-	-	$CL = (\theta_{CL,a} \times GFR^{1.2}) / (\theta_{CL,b}{}^{1.2} + GFR^{1.2})$ $V_1 = \theta_V \times (BW/70)$
Bos JC et al.[14]	1	CrCl	Mixed	3.44	27.1	22.4	39.4	-	-	-	-	$CL = 3.44 \times (1 + 0.00925 \times (CrCl - 92))$
Hodiamont et al.[15]	2	IBW, ALBM	Proportional	2.12	49.1	23.9	45.7	1.95	-	18.1	-	$CL = 2.11 \times (IBW/70)^{0.75}$ $V_1 = 21.2 \times (IBW/70) \times (ALBM/22)^{-0.833}$
Hodiamont et al. [16]	2	-	Mixed	1.63	54.8	8.67	45.7	0.943	-	6.78	-	$CL = 1.63 \times (BW/70)^{0.75}$ $Q = 0.943 \times (BW/70)^{0.75}$ $V_1 = 8.67 \times (BW/70)$

Cmpt, compartment; *Cov*, covariates, *CL*, clearance; *V*, central volume of distribution; V_1, volume of distribution of the first compartment; *Q*, inter-compartmental clearance; V_2, volume of distribution of the second compartment; *IIV*, interindividual variability, *ALBM*, Albumin, *CrCl*, creatinine clearance; *GFR*, glomerular filtration rate, *BW*, bodyweight, *IBW*, ideal bodyweight.

Table S3. Typical value of PK parameters and variability used during external evaluation and following the re-estimation of PopPK models.

Model	External evaluation								Following re-estimation							
	CL	IIV_{CL} (%)	V or V_1	$IIV_{V\ or\ V1}$ (%)	Q	IIV_Q (%)	V_2	IIV_{V2} (%)	CL	IIV_{CL} (%)	V or V_1	$IIV_{V\ or\ V1}$ (%)	Q	IIV_Q (%)	V_2	IIV_{V2} (%)
Rea RS et al. [13]	1.45	83.7	53	64.4	-	-	-	-	2.26	60.7	21.7	42.8	-	-	-	-
Bos JC et al. [14]	5.7	74	19	49	-	-	-	-	4.01	27.0	22.4	39.4	-	-	-	-
Hodiamont et al. [15]	1.90	42.5	21.2	17.2	1.96	-	18.4	-	2.12	49.1	23.9	45.7	1.95	-	18.1	-
Hodiamont et al. [16]	2.3	75.0	21.6	27.0	1.3	-	10.2	-	1.63	54.8	8.67	45.7	0.943	-	6.78	-

CL, clearance; *V*, central volume of distribution; V_1, volume of distribution of the first compartment; *Q*, inter-compartmental clearance; V_2, volume of distribution of the second compartment; *IIV*, interindividual variability.

Table S4. Bootstrap Results of the Re-estimated Model from Rea et al.

	Bootstrap of re-estimated model	
Parameter	Mean	95% CI
ϴCL,a (L/h)	9.74	9.62 – 9.87
ϴCL,b	136	134 – 138
ϴV (L)	21.8	21.7 – 21.9
Interindividual variability (IIV)		
CL^a (CV%)	35.9	35.6 – 36.2
CL^b (CV%)	16.6	16.3 – 16.9
V (CV%)	27.9	27.5 – 28.2
Residual Error		
Additive (mg/L)	0.279	0.275 – 0.282
Proportional (CV%)	34.4	34.1 – 34.6

Cmpt, compartment; *Cov*, covariates, *CL*, clearance; *V*, central volume of distribution; V_1, volume of distribution of the first compartment; *Q*, inter-compartmental clearance; V_2, volume of distribution of the second compartment; *IIV*, interindividual variability, *ALBM*, Albumin, *CrCl*, creatinine clearance; *GFR*, glomerular filtration rate, *BW*, bodyweight, *IBW*, ideal bodyweight.

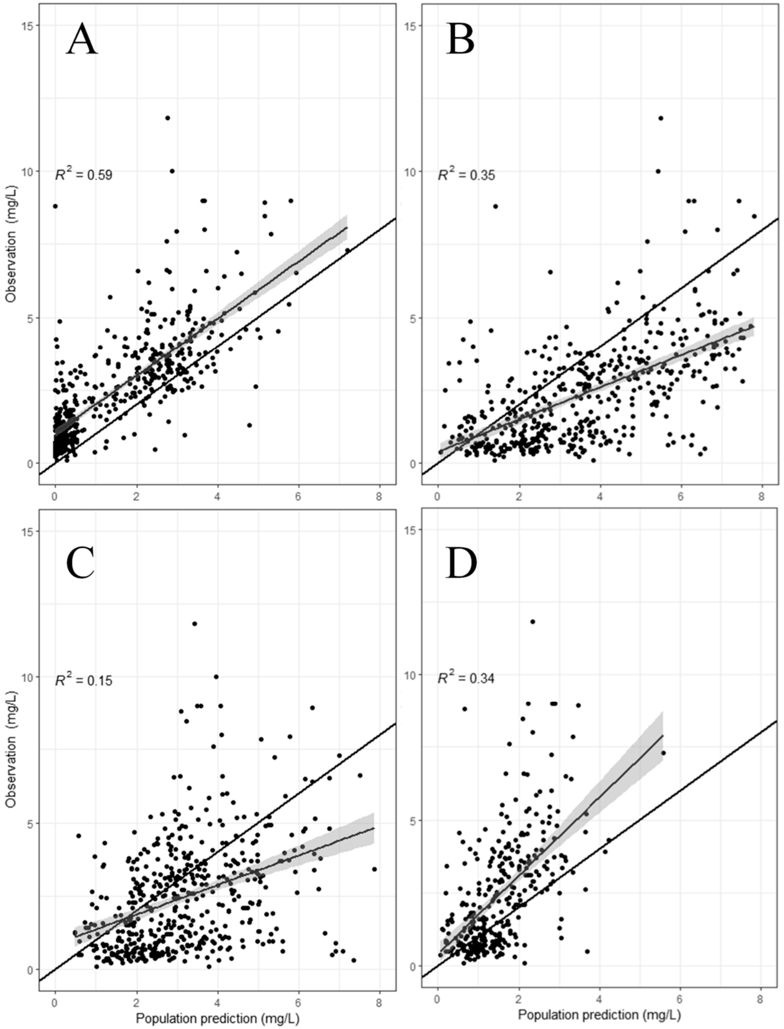

Figure S1. Population-predicted concentration versus observed concentrations for gentamicin models. A. Bos et al. [14], B. Hodiamont et al. [15], C. Rea et al. [13], D. Hodiamont et al [16]. Black line with shaded area represents the trendline from the scatter points.

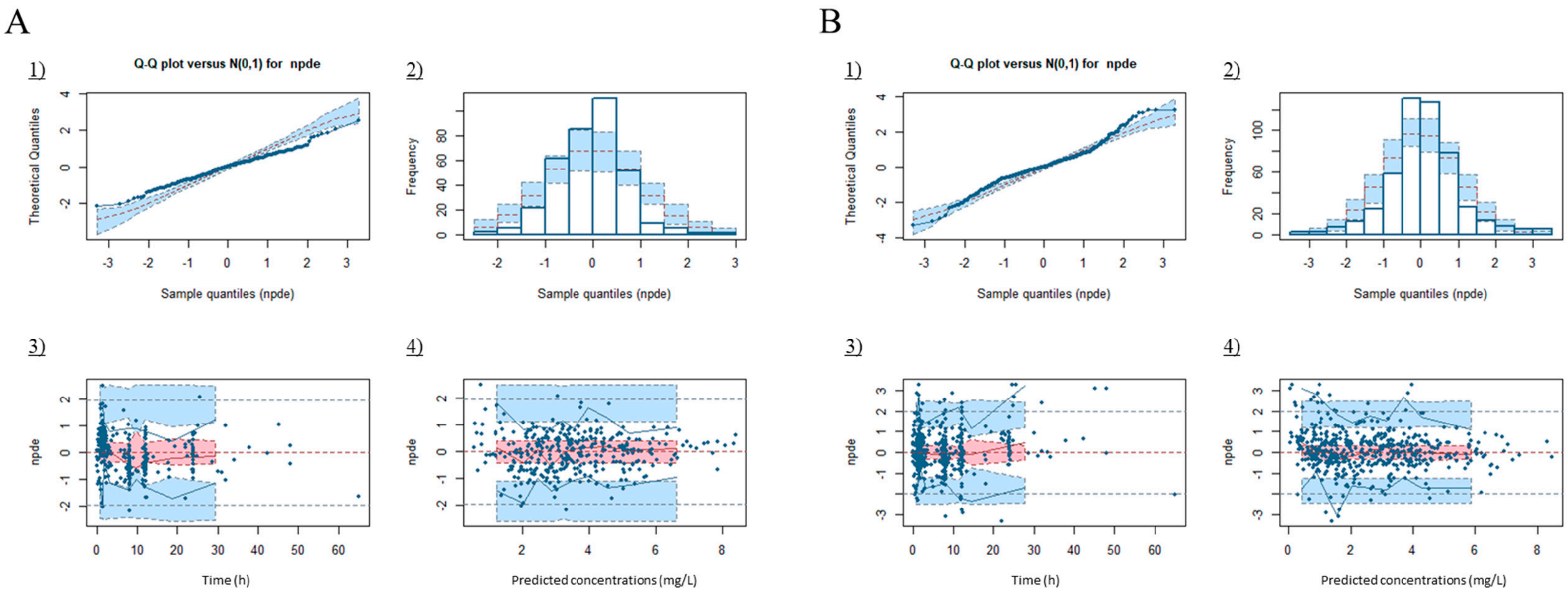

Figure S2. Normalized prediction distribution errors (NPDE) plots of (A) the external evaluation and (B) following model re-estimation for Rea et al. 1) Q-Q plot of the NPDE, 2) Histogram of the NPDE, 3) NPDE versus time, 4) NPDE versus predicted concentrations.

Table S5. Probability of target attainment of C_{max}/MIC >8 on the 3rd dose based on different MIC values and MDD and ODD dosing regimens of gentamicin.

MIC	3 mg/kg/day (%)			4 mg/kg/day (%)			5 mg/kg/day (%)		
	1 mg/kg/q8h	1.5 mg/kg/q12h	3 mg/kg/q24h	1.33 mg/kg/q8h	2 mg/kg/q12h	4 mg/kg/q24h	1.67 mg/kg/q8h	2.5 mg/kg/q12h	5 mg/kg/q24h
0.25	91.6	94.6	99.2	95.5	97.7	99.1	97.7	98.1	99.4
0.5	57.3	75.7	93.8	77	88.6	97.2	87.3	93.5	98.0
1	8.6	20.9	62.3	25.1	47.0	80.7	41.0	64.8	90.5
2	0.1	0.3	13.3	0.8	3.5	30.7	2.6	13.2	51.3
4	0	0	0.5	0	0	2.0	0	0	6.5

Table S5. Continued.

MIC	6 mg/kg/day (%)			7 mg/kg/day (%)			8 mg/kg/day (%)		
	2 mg/kg/q8h	3 mg/kg/q12h	6 mg/kg/q24h	2.33 mg/kg/q8h	3.5 mg/kg/q12h	7 mg/kg/q24h	2.66 mg/kg/q8h	4 mg/kg/q12h	8 mg/kg/q24h
0.25	99.1	98.6	99.3	99.0	99.4	100.0	98.7	99.5	99.4
0.5	92.6	96.7	98.5	95.5	97.5	99.1	95.8	98.3	99.3
1	57.9	75.4	93.2	69.2	82.5	96.4	76.2	87.2	97.3
2	8.1	21.4	61.7	14.5	33.5	74.8	24.4	44.7	82.8
4	0.1	1	14.1	0.4	2.5	22.5	0.8	4.6	33.8

Table S5. Continued.

MIC	10 mg/kg/day (%)			12 mg/kg/day (%)		
	3.33 mg/kg/q8h	5 mg/kg/q12h	10 mg/kg/q24h	3 mg/kg/q8h	4 mg/kg/q12h	12 mg/kg/q24h
0.25	99.1	99.5	99.7	99.7	99.6	99.9
0.5	97.4	98.8	99.5	97.9	99.5	99.8
1	87.4	94.1	98.6	90.5	96.4	98.8
2	37.7	62.2	89.5	54.5	77.4	94.2
4	2.7	11.3	49.4	8.2	20.2	65.8

MIC, Minimum inhibitory concentration; *q8h,* every 8 hours; *q12h,* every 12 hours, *q24h*, every 24 hours.

Table S6. Probability of target attainment of C_{max}/MIC >10 on the 3rd dose based on different MIC values and MDD and ODD dosing regimens of gentamicin.

MIC	3 mg/kg/day (%)			4 mg/kg/day (%)			5 mg/kg/day (%)		
	1 mg/kg/q8h	1.5 mg/kg/q12h	3 mg/kg/q24h	1.33 mg/kg/q8h	2 mg/kg/q12h	4 mg/kg/q24h	1.67 mg/kg/q8h	2.5 mg/kg/q12h	5 mg/kg/q24h
0.25	84.7	91.3	98.2	92.6	96.9	98.7	92.6	97.4	99.4
0.5	37.5	60.1	89.2	61.1	78.8	94.2	61.1	88.4	97.4
1	2.1	8.5	46.2	9.5	27.8	67.2	22.4	44.7	82.0
2	0	0	5.9	0	0.6	15.5	0.2	4.3	32.8
4	0	0	0	0	0	0.8	0	0	2.0

Table S6. Continued.

MIC	6 mg/kg/day (%)			7 mg/kg/day (%)			8 mg/kg/day (%)		
	2 mg/kg/q8h	3 mg/kg/q12h	6 mg/kg/q24h	2.33 mg/kg/q8h	3.5 mg/kg/q12h	7 mg/kg/q24h	2.66 mg/kg/q8h	4 mg/kg/q12h	8 mg/kg/q24h
0.25	96.1	98.4	99.0	97.8	98.9	99.8	98.3	99.3	99.4
0.5	77.1	92.8	97.8	85.7	96.0	98.2	92.0	97.1	98.9
1	22.4	60.8	86.5	38.7	73.0	92.5	61.0	77.8	94.6
2	0.2	10.2	47.0	2.2	18.2	60.1	12.0	26.1	71.2
4	0	0.3	6.1	0	0.8	11.3	0	1.1	19.1

Table S6. Continued.

MIC	10 mg/kg/day (%)			12 mg/kg/day (%)		
	3.33 mg/kg/q8h	5 mg/kg/q12h	10 mg/kg/q24h	3 mg/kg/q8h	4 mg/kg/q12h	12 mg/kg/q24h
0.25	98.9	99.5	99.7	99.6	99.6	99.9
0.5	96.2	98.3	99.1	97.1	98.7	99.6
1	76.0	89.2	97.1	83.7	92.6	98.5
2	20.5	43.7	80.6	35.6	59.7	90.0
4	0.4	3.9	31.6	2.7	9.1	46.3

MIC, Minimum inhibitory concentration; *q8h*, every 8 hours; *q12h*, every 12 hours, *q24h*, every 24 hours.

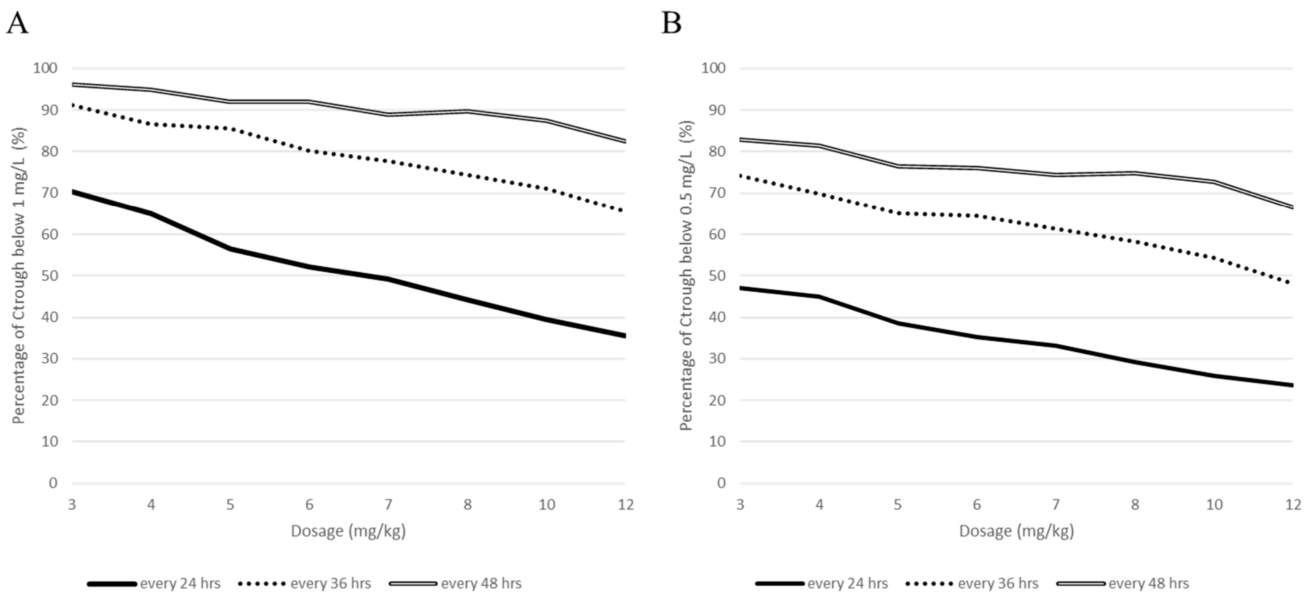

Figure S3. Percentage of C_{trough} following different dosage regimens (every 24 hours, every 36 hours and every 48 hours) and dosing interval below **A.** 1 mg/L **B.** 0.5 mg/L.

Table S7. Percentage of C_{trough} below 1 mg/L or 0.5 mg/L before the 4th administration following different dosage regimens and dosing interval below.

C_{trough}	3 mg/kg (%)					4 mg/kg (%)				
(mg/L)	Q8h	Q12h	Q24h	Q36h	Q48h	Q8h	Q12h	Q24h	Q36h	Q48h
< 1	4.8	18.9	70.3	91.1	96.1	3.6	14.3	65.0	86.7	95.0
< 0.5	1.5	8.9	47.0	74.2	82.8	1.7	6.8	45.0	69.9	81.5

Table S7 Continued.

C_{trough} (mg/L)	5 mg/kg (%)					6 mg/kg (%)				
	Q8h	Q12h	Q24h	Q36h	Q48h	Q8h	Q12h	Q24h	Q36h	Q48h
< 1	2.9	9	56.5	85.6	92.0	2.2	8.8	52.1	80.3	92.0
< 0.5	1.2	4.5	38.5	65.1	76.4	0.9	3.7	35.2	64.5	76.0

Table S7 Continued.

C_{trough} (mg/L)	7 mg/kg (%)					8 mg/kg (%)		
	Q8h	Q12h	Q24h	Q36h	Q48h	Q24h	Q36h	Q48h
< 1	2.5	6.9	49.3	77.7	89.0	44.2	74.3	89.8
< 0.5	1.3	3.6	33.1	61.3	74.4	29.3	58.3	74.8

Table S7 Continued.

C_{trough} (mg/L)	10 mg/kg (%)			12 mg/kg (%)		
	Q24h	Q36h	Q48h	Q24h	Q36h	Q48h
< 1	39.6	71.1	87.5	35.6	65.5	82.4
< 0.5	25.9	54.4	72.8	23.6	48.1	66.6

C_{trough}, concentration before the 4th administration; *MIC*, Minimum inhibitory concentration; *q8h*, every 8 hours, *q12h*, every 12 hours, *q24h*, every 24 hours; *q36h*, every 36 hours; *q48h*, every 48 hours .
